# A suspended polymeric microfluidic sensor for liquid flow rate measurement in microchannels

**DOI:** 10.1038/s41598-022-06656-z

**Published:** 2022-02-16

**Authors:** Fatemeh Mohammadamini, Javad Rahbar Shahrouzi, Mitra Samadi

**Affiliations:** Faculty of Chemical Engineering, Sahand University of Technology, Sahand New Town, Tabriz, Iran

**Keywords:** Chemical engineering, Mechanical engineering, Fluid dynamics, Techniques and instrumentation

## Abstract

In this study, a microfluidic cantilever flow sensor was designed and manufactured to monitor liquid flow rate within the range of 100–1000 µl/min. System simulation was also performed to determine the influential optimal parameters and compare the results with experimental data. A flowmeter was constructed as a curved cantilever with dimensions of 6.9 × 0.5 × 0.6 mm^3^ and a microchannel carved with a CO_2_ laser inside the cantilever beam. The fabrication substance was Polydimethylsiloxane. Different flow rates were injected using a syringe pump to test the performance of the flowmeter. Vertical displacement of the cantilever was measured in each flowrate using a digital microscope. According to the results, the full-scale overall device accuracy was up to ± 1.39%, and the response time of the sensor was measured to be 6.3 s. The microchip sensitivity was 0.126 µm/(µl/min) in the range of measured flow rates. The sensor could also be utilized multiple times with an acceptable error value. The experimental data obtained by the constructed microchip had a linear trend (R^2^ = 0.995) and were of good consistency with simulation results. Furthermore, according to the experimental and the simulation data, the initially curved cantilever structure had a higher bending and sensitivity level than a perfectly straight cantilever construction.

## Introduction

Over the recent decades, microfluidic technology has been widely used in various applications. Thanks to the possibility of using a small amount of sample, this kind of sensor has captured interest as a useful device to perform operations, including separations, reactions, or the detection of various objects, such as materials and particles. This technology has also been employed in biomedical applications, e.g., drug delivery, DNA/Gene analysis, and diagnosis of disease by lab-on-a-chip (LOC), or organ-on-a-chip, microreactors, and micro total analysis systems (µTAS)^1^. This technology also applies to commercial products, including home pregnancy testing, virus fast testing (e.g., HIV; Herpes Simplex; COVID-19; and Hepatitis A, B, and C), and blood glucose detection^2,3^.

A stable liquid flow in the microfluidic system is crucial since flow variations directly induce product failure^1,4,5^, especially in applications, such as particle sorting and separation, flow cytometry, flow mixing, chemical synthesis, and polymerase chain reaction (PCR)^6^. Coriolis mass flowmeter and precision syringe pump are often used for this purpose. However, they are limited by bulk size, high cost, and complex connection to microchips^7^. Thus, micro-electro-mechanical systems (MEMS) have been proposed by researchers as a means to miniaturize flow sensors. Because of their low power consumption, high precision, short response time, portability, and cost-effectiveness, MEMS-based flow sensors are ideal to be used in microfluidic systems^1^.

MEMS flow sensors are either thermal or non-thermal. Thermal flow sensors are the most commercially available devices for use in microfluidic systems because of their high sensitivity^3^. Kim et al.^8^ determined the liquid flowrate by heating and sensing electrodes to measure the thermal distribution inside the microchannel. Zhao et al.^7^ developed a thermal time-of-flight sensing microchip relying on thermal excitations. Due to the high thermal diffusivity, heat loss through microchannels might be hazardous to particular applications, such as living cells, causing the sensor to respond improperly^9^. Non-thermal flow sensors are also available, including flowrate measurement based on changes in the conductivity of a microwave resonator^4^, an electrochemical sensor measuring ion conductivity changes^9^, micro bubble image velocimetry using gas bubbles as a tracer^10^, an optofluidic flowmeter using micro/nanofibers^11^, and a digital volume dispensing system working by electrically detecting the frequency of droplet generation^12^.

Mechanical flow sensors comprising a moving device, such as a cantilever, spring, or diaphragm, are also often used to determine the flow rate. These sensors can be simply designed, are cost-effective, and operate in a simple manner^1^. In recent years, cantilever-based flow measurement has captured a lot of interest. A cantilever bends when a force applies to it. A cantilever structure consists of a moveable and a fixed part. Accordingly, a cantilever is made of a thin membrane, a beam, or a plate with one side attached to a support and the other free to bend. A cantilever flow sensor is a type of pressure flowmeter in which flow is in contact with the surface of the cantilever, causing the cantilever to bend in the direction of acting force. In order to calculate the flow rate using a calibration curve, deflection must be measured using optical, piezoelectric, piezoresistive, or capacitive methods^13^. As an example of flow measurement with a cantilever structure, Naveen et al.^14^ created a stainless-steel cantilever along the pipeline to measure the flow rate at high volumes. Image analysis was employed to determine the cantilever's deflection. Hamdollahi et al.^15^ also studied a cantilever-based air flowmeter that calculates gas flow rate through image processing.

Due to its simple structure and function, the cantilever is of the favorable capability to be used as a flow sensor in microfluidic systems. Microcantilevers are reported to be used in microfluidic systems, including analyte detection^16^, biosensing of DNA^17^, detection of heavy metal ions^18^, and viscometers^19^. In this regard, some initial works were carried out in the microfluidic systems flow metering field. Aiyar et al.^20^ developed a micromachined airflow sensor in aerial vehicles to perceive an artificial skin by measuring air velocity. In their work, polyimide film (Kapton) was used as the base material of the sensor. Quist et al.^21^ developed a piezoresistive cantilever sensor to measure flowrate and viscosity in microchannel and refined the beam structure to measure fluid speed in terms of cm/s. Lien et al.^22^ also manufactured a fiber-optic cantilever flow sensor for liquid flow in a microchannel with high sensitivity and dynamic range of 0–1500 µl/min. Afterward, as technology progressed, more intricate forms of flowmeters were proposed to measure flow characteristics in microchannels. Ju et al.^23^, for instance, studied a flow-induced vibrating sensor for flow monitoring. The sensor was an etched fiber-optic cantilever with a diameter of 9 µm for flow sensing inside a microchannel with dimensions of 100 × 70 µm^2^, which could also be used to analyze properties of non-Newtonian liquids. Sanati Nezhad et al.^5^ constructed a multilayer polydimethylsiloxane (PDMS) microcantilever incorporated within a microfluidic device and used an optic microscope to assess the cantilever bending. In another work, Neoth et al.^6^ designed two microcantilever flow sensors made of SU-8 and SiN materials to indicate the effect of surface holes on cantilever deformation using a position-sensitive detector (PSD) system that monitors the ending level. Cheri et al.^1^ manufactured a cantilever-based optofluidic flow sensor integrated with optical fibers for real-time flowrate measurement. Instead of a time-consuming multilayer lithography method, the fabrication process was carried by casting method using laser engraving of PMMA. Wang et al.^24^ designed a free-standing piezoresistive-based cantilever to measure airflow velocity. In their study, the resistance of the platinum pizeoresistor affected the cantilever beam as the airflow changed. Platinum resistance was also determined using an external LCR meter. Verlinden et al.^25^ proposed a two-channel microfluidic AFM cantilever to enable multiple reagents dosing inside another liquid environment as an application to highlight the importance of flow metric in (bio) chemical reactions. This device was capable of controlling flow rates ranging from femtoliters to pico-liters per second. Garrett et al.^26^ also proposed a biocompatible microfluidic flow sensor to assess the cerebrospinal fluid shunt as a functional sensor in the medical industry. This PDMS cantilever could detect the flow rates within the range of 20–120 mL/h, which is the particular range for cerebral spinal fluid.

Fabrication and characterization of a polymeric micro cantilever liquid flow sensor were carried out in this study. The design of this sensor was inspired by suspended polymeric microfluidic (SPMF) systems, which are commonly employed for particle detection in biological applications^27^ or monitoring the density and viscosity of fluids^19^. In this field, the cantilever is not exposed to flow, but rather, the liquid passes through the embedded microchannel inside the cantilever, and the flow force acting on the interior walls causes it to bend. Before the fabrication process, a 3D computational fluid dynamic (CFD) simulation was performed to design an appropriate size for microchannel and cantilever construction. The simulation results on the system are required to determine the dynamic flowrate range within which the cantilever has a linear response^28^. In order to skip the conventional lithography steps, the microchannel was encarved by a CO_2_ laser on a prepared PDMS polymeric layer to fabricate the sensor. In this research, the effect of the initially curved geometry of the cantilever on flowmeter performance was assessed and compared with the initially straight one. Afterward, a comparison was made between simulation results and experimental data, and conformity degree was assessed.

## Fabrication and experimental process

### Simulation and design

**Figure 1:**
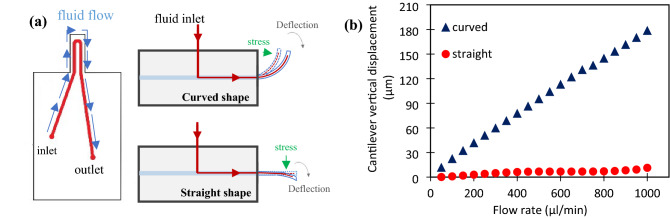
(**a**) The fundamental operational process of the flowmeter. (**b**) Comparison of the simulations results for the cantilever displacement in curved and straight shapes.

As mentioned earlier, the change in the direction of the fluid inside the microchannel applies force on the cantilever and makes it deform. The fundamental operation of the flowmeter is shown in Fig. 1a. The force on the cantilever wall acts in the vertical and horizontal directions. Fluid pressure is the source of the vertically applied force, while the horizontal force stems from shear stress^29^. However, cantilever deformation is governed by the Stoney equation indicated below^13^.

1 $$\updelta =\frac{3{l}^{2}(1-\upnu )}{\mathrm{E}{t}^{2}}\mathrm{\Delta \sigma }$$

2 $$F=\mu \Delta u+A\Delta P={k}_{m}\delta$$

3 $${k}_{m}=\frac{3EI}{{l}^{3}}$$

where $\delta$ is the vertical cantilever deformation, E and $\nu$ respectively are Young’s modulus and the Poisson’s coefficient of the substrate used in the beam structure. $\sigma$ indicates the normal stress applied to the material. F is the applied force to the cantilever, $\mu$ is fluid viscosity, $\Delta u$ indicates the velocity change, A is the surface of the cantilever perpendicular to the fluid, and ${k}_{m}$ is the stiffness factor for a cantilever with the length of *l* and thickness of *t*.

The sensitivity of a cantilever sensor is determined by its physical dimensions and mechanical properties of the material it is made of^30^. The system's preliminary design was carried out by simulation to achieve appropriate dimensions for constructing a high-sensitivity sensor in the range of the required flow rate. High sensitivity here refers to high cantilever deflection over the required flowrate range. The parameters that affect the cantilever deflection must be optimized, such as material type, cantilever and microchannel dimensions, microchannel geometry, and placement coordinates inside the cantilever. Accordingly, a coupled solid mechanic and laminar flow phenomena were used to simulate the interaction between fluid and solid structure in COMSOL Multiphysics 5.3. Assumptions used to simplify the problem include steady-state, fully developed, laminar fluid flow, non-slip boundary condition, the square cross-section perpendicular to fluid, and smooth wall surface. Mass and momentum balances for fluid and solid elastic equations at steady-state conditions are expressed as follows:

4 $$\frac{\partial \rho }{\partial t}+\nabla \bullet (\rho u)=0$$

5 $$\rho (u\bullet \nabla u)=-\nabla \bullet [-pI+\mu (\nabla u+(\nabla u{)}^{T}]+F$$

6 $${F}_{\nu }=-\nabla \bullet \sigma$$

where $\rho (\mathrm{kg}/{\mathrm{m}}^{3})$ represents fluid density, *u* (m/s) is fluid velocity vector, *p* (Pa) is the pressure, *I* is the identity matrix, $\eta \left(\mathrm{kg}/\mathrm{m s}\right)$ is the fluid viscosity, *F* is the acceleration force density, ${F}_{\nu }$ is the applied force to solid per space unit, and $\sigma$ (N/m^2^) is the total stress applied to microchannel interior wall. The equations were solved using the boundary conditions listed as follows.

- The fluid stable inlet flow rate was in the range of 100–1000 µl/min.
- Relative outlet fluid pressure is equal to 0 due to entering the atmospheric environment.
- No-slip boundary condition for microchannel interior walls
- Definition of fixed constraint for microcantilever at the inlet base
- Definition of the cantilever as a linear elastic material

The sensor performance depends on the amount of total displacement taking place in the free end of the cantilever due to different flow rates. In this regard, the cantilever bending level was optimized in the desired range by software. After simulation and investigation of all factors affecting cantilever bending, the optimal value for each parameter was obtained, as shown in Table 1. Furthermore, according to simulation results, the construction of a curved cantilever plays a determining role in increasing bending. For this purpose, the effect of the different initial shapes (curvature) of the cantilever on the bending values was compared, as shown in Fig. 1b. According to this diagram, the bending value of the curved cantilever is greater than that of the straight one for the same range of flowrate. Therefore, fabricating a cantilever with a curved construction is preferable in increasing the sensitivity of the sensor. It is worth mentioning that raising the curvature value of the cantilever has a positive effect on the deflection.

**Table 1:** The values of parameters and dimensions for cantilever fabrication.

Dimensions (mm)	Cantilever	Length	Width	Depth
		9	0.4	0.6
	Microchannel	7.8	0.6	0.1–0.15
Material properties^31^	PDMS	Young’s modulus	Poisson’s ratio	density
		1400 kPa	0.49	970 kg/m^3^
Microchannel tip shape	Circular			
Placement coordinate	At a distance of 0.4 mm from the bottom face of the cantilever			

### Preparation of cantilever and microchannel

**Figure 2:**
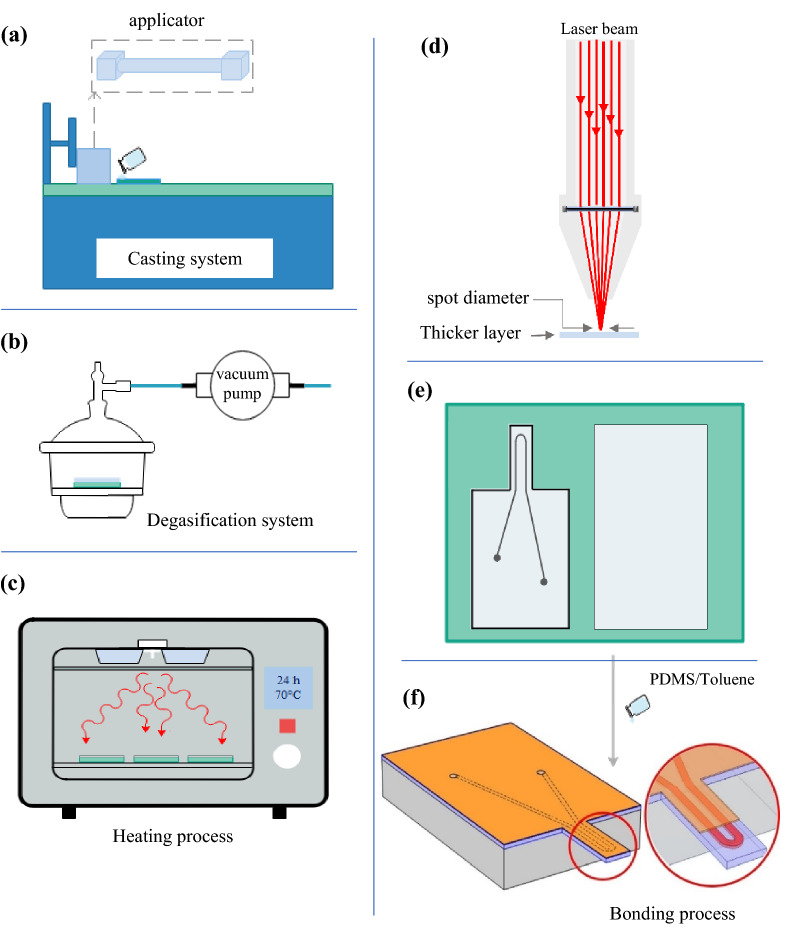
The fabrication process of the microchip: (**a**) smooth layer casting process, (**b**) degasification step, (**c**) heating process, (**d**) CO_2_ laser engraving step, (**e**) prepared PDMS films, and (**f**) schematic representation of the fabricated microcantilever sensor.

**Figure 3:**
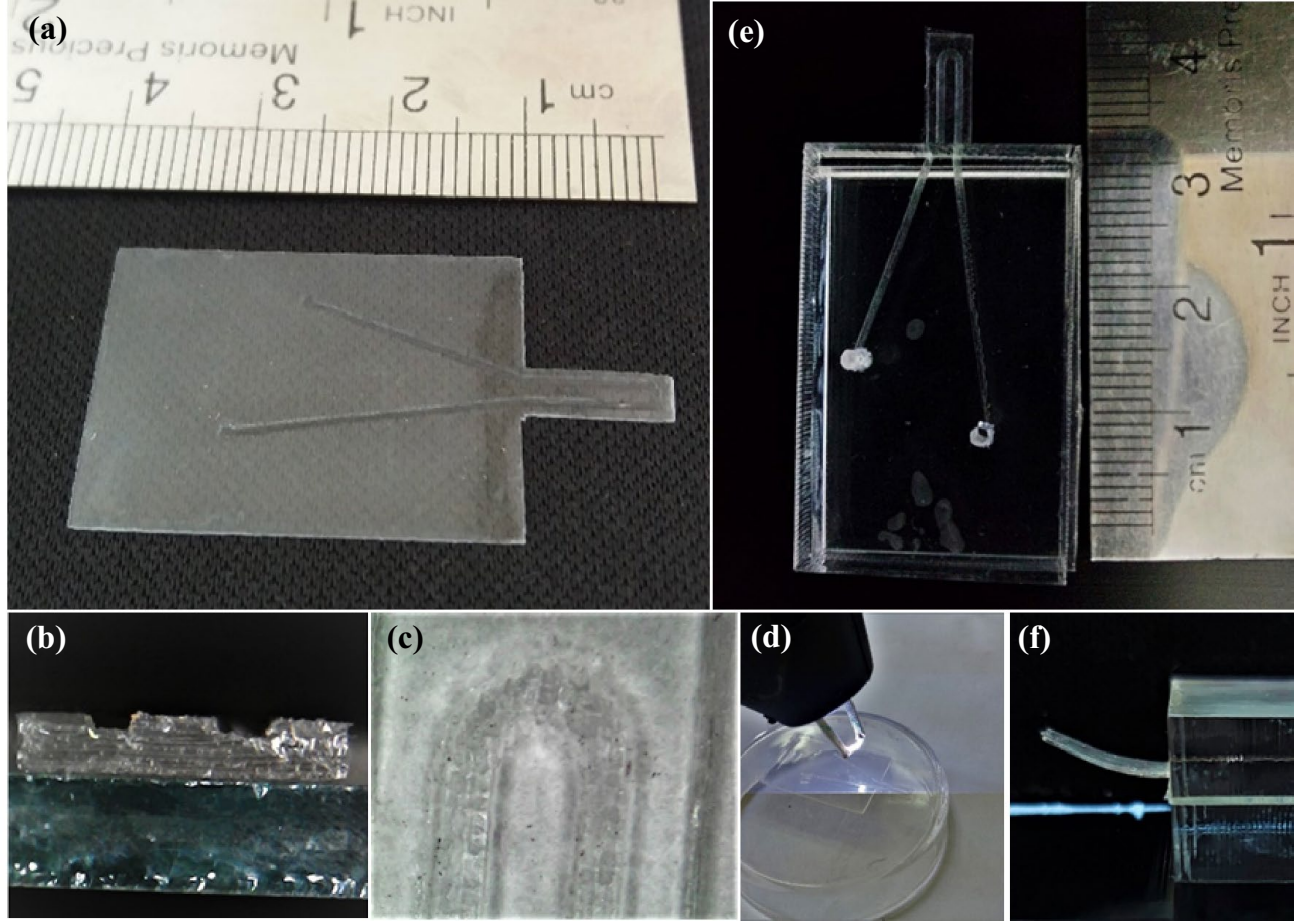
(**a**–**c**) Different cross views of PDMS film after engraving and cutting by CO_2_ laser. (**d**) Polymer surface treatment through corona plasma before bonding two layers, (**e**) and (**f**) final shape of the sensor. Photos were taken by HiView v. 1.4 software (http://www.hvscam.com).

Figure 2 illustrates the schematic of the microcantilever fabrication process. In order to to provide a polymeric layer for cantilever sensor fabrication, a mixture of Polydimethylsiloxane (PDMS) was prepared with a ratio of 1:10 (cross-linker:base) and poured into a glass base to cast a smooth layer with applicator device. After the degasification process through a vacuum pump (GEC BS500011, England), the casted films were baked at 70 °C for 24 h. Then, the resulting polymeric film was peeled off from the glass. In order to fabricate a cantilever with an overall thickness of 600 µm, two films were prepared with thicknesses of 100 and 500 µm. Afterward, the shape designed for microchannel and cantilever in optimal dimensions was drawn with CorelDRAW v.X7 software and prepared for engraving on a polymer layer with a thickness of 500 µm by CO_2_ laser (Yuemimg Laser PM1380, China). Photos of the engraved PDMS film are shown in Fig. 3a–c.

### Bonding process

After the engraving process, the prepared films were washed with isopropanol to remove extra particles from lasering. Then, clean films were prepared for bonding to form the microchannel. This step was carried out by applying an adhesive layer, which was prepared by mixing PDMS and toluene with a ratio of 1:2 on both polymeric films^31^ (see Fig. 2f). It should be noted that plasma treatment on the surface increases the hydrophilic level of PDMS films and improves surface coverage with the adhesive layer^32,33^. Therefore, before coating the films with glue, one of them was treated with corona plasma radiation. After applying adhesive on both 100 and 500 μm films, they were gently placed on each other, heated for 15 min at 70 °C for initial bake, and left at room temperature for 24 h without any displacement. Afterward, the films were completely stuck to each other and were ready for fluid injection. In order to fix one side of the cantilever, the polymeric microchip was sandwiched between PMMA sheets except for the cantilever part. For this purpose, an adhesive of the same type was prepared, and plasma corona irradiation was used. A vacuum pumping step was also required to remove the trapped air between PDMS and PMMA sheets. Figure 3d–f demonstrates the corona treatment of PDMS surface and final micro flow sensor after the fixation process.

### Experimental setup

**Figure 4:**
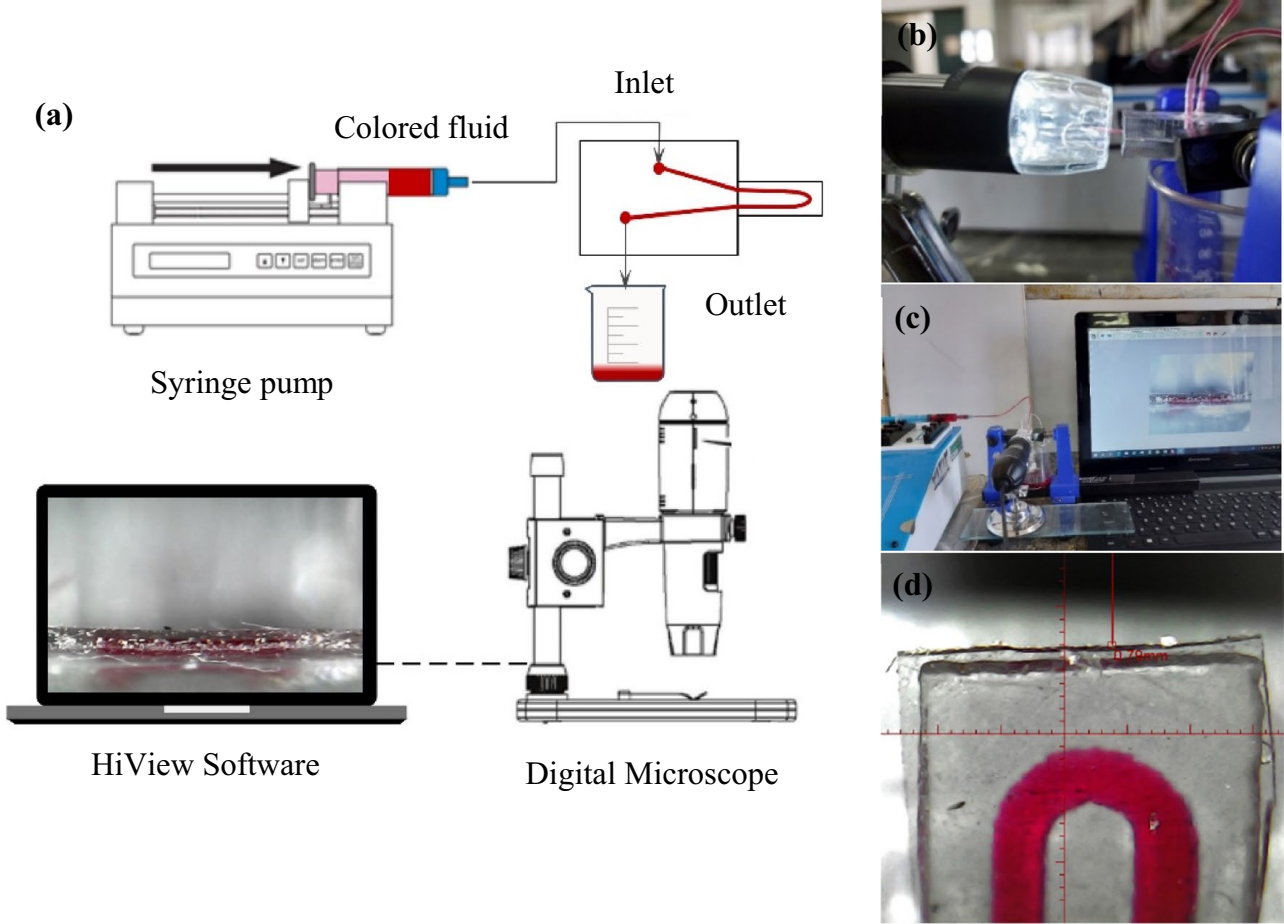
(**a**) Schematic of the experimental setup for flow measuring by microcantilever, (b), (c) photography system, and (**d**) measuring the vertical displacement of the curved cantilever by HiView v.1.4 software (http://www.hvscam.com).

The experimental setup is demonstrated in Fig. 4a–c. In order to inject liquid into the microchannel, a high precise calibrated syringe pump (SP125, Iran) was employed. Silicon tube with a diameter of 1 mm were used to connect the chip to the syringe pump. In addition, a small piece of a rigid Teflon tube with a diameter of 1 mm was used to connect the silicone tube and the PMMA sheet. It is worth noting that the connections were sealed via waterproof glue to prevent fluid leakage from the connections. A digital CMOS microscope (1600x, China) was applied to measure the vertical bending value of the microcantilever. The microscope was placed perpendicular to the cantilever, and the measurement was performed through HiView v.1.4 software. An example of the measuring process is shown in Fig. 4d.

## Results and discussion

### Repeatability of responses

**Figure 5:**
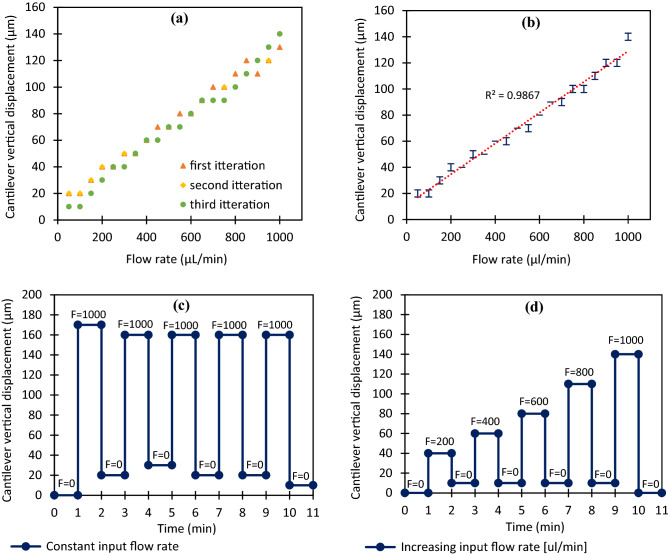
The value of vertical displacement of the cantilever (**a**) the observed results for three different tests and (**b**) linearization and measured error after three times of testing. (**c**, **d**) The sensor reversibility to initial state in consecutive use of micro flowmeter in two modes of constant and variable input flowrates.

Bending performance is the critical feature of a sensor after it is used several times. Therefore, the repeatability of the flow sensor was examined by comparing the vertical deformation of the cantilever at multiple applications. Figure 5a, b shows the vertical deformation data for three different tests performed on one day under the same laboratory conditions and at equal time intervals to assess the repeatability characteristic of the flowmeter. According to the data, the average standard deviation of sensor response was approximately 1.9. It is worth mentioning that the instrument cannot accurately measure the amount of deformation at flow rates lower than 200 L/min due to the low resolution of the displacement measurement camera. Besides, the test was repeated after one week to study the effect of time interval on the vertical displacement of the cantilever under the same laboratory conditions. According to this comparison, as the number of times, the microchip is used to measure flow rate increases, the final value of vertical displacement significantly changes and deviates from the original value, especially at higher flow rates. Accordingly, it can be deduced that the sensor requires re-calibration for long-term uses.

### Flowmeter reversibility

Another essential feature of mechanical sensors is their mechanical ability to return to their original position after multiple uses. In order to study this feature of the sensor, two independent tests were conducted: constant input flowrate and increasing input flowrate. In either case, the initial position of the cantilever pertinent to the vertical axis was measured using a digital camera. The cantilever displacement due to the force exerted by fluid was measured at the beginning of fluid injection into the microchannel, and its value was recorded in one minute. The cantilever was then returned to its original state by terminating the fluid injection into the microchannel. Then, after a one-minute pause, the measurement procedure was repeated in the same way. This experiment was carried out on the manufactured sensor in two different constant and increasing input flow rate modes. The results are presented in Fig. 5c, d, respectively. According to the results of these tests, the probability of sensor reversibility may deviate up to 15% from the initial position at the constant flow rate, especially at higher input flow rate values. This deviation was reduced to 5% as the input flow rate increased. As an elastic polymer, PDMS shows excellent robustness against fatigue. According to the reported result in Figs. 5c, d, only a small amount of tension remained inside the polymeric layers of the cantilever after the sensor was used several times. Thereby, the robustness of the sensor against fatigue is acceptable. However, the cantilever will be back to its original position if enough time is given. While using this sensor, a serious failure is that the bond between the bonded layers might break due to the pressure of the fluid created by the over ranged fluid flow. In this case, the bond between the layers will break, and the leakage will take place. Consequently, the measurement will fail.

### Accuracy

**Figure 6:**
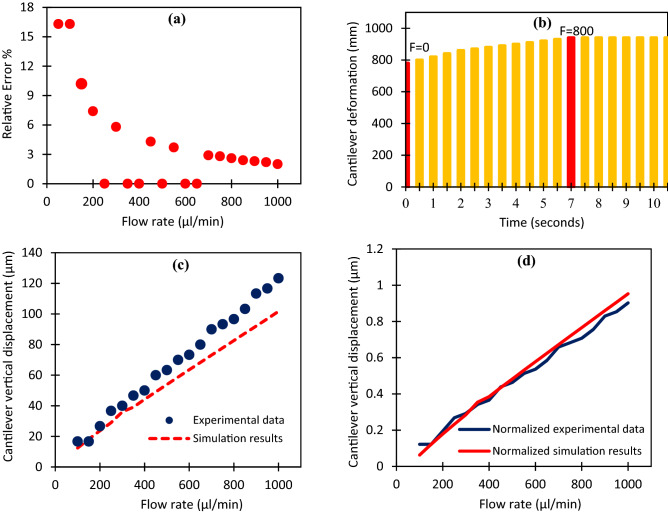
(**a**) Relative error percentage of microcantilever deformation in different flow rates. (**b**) Cantilever vertical deformation versus time for an input flow rate of 800 µl/min. Comparison between changes in (**c**) experimental and simulation results and (**d**) normalized data at different input flowrates.

It is feasible to quantify the amount of device error in each repetition relative to the average performance using the data gathered from numerous usages to determine the accuracy of the sensor. For this purpose, two types of relative and full-scale error were calculated. The relative error result is shown in Fig. 6a. According to the results, at low flow rates, the error of the device is much more significant than that at high flow rates, which can be attributed to the low accuracy of the microscope in measuring the displacement at low flow ranges. Eventually, the average full-scale error percentage of the sensor was determined to be ± 1.39%.

### Response time

The shorter the response time of a flowmeter, the more instantaneously it can be used. System response time refers to the time it takes from the cantilever to be stable in a fixed position after turning on the syringe pump. In general, the degree of material elasticity used in manufacturing a cantilever sensor can affect its response time, in addition to the intensity of the input fluid. Therefore, using a more elastic material for manufacturing a cantilever sensor causes rapid position changes. Ultimately, after analyzing system response time at different input flow rates, the average response time of the flowmeter was determined to be 6.3 s. Figure 6b illustrates the vertical deformation value of the cantilever at 800 µl/min input flow rate.

### Simulation results

The results of the comparison between the average value of cantilever vertical displacement at different flow rates and the simulation results for the same microchip are remarkable, as shown in Fig. 6c. According to this result, as the input flow rate increases, the deviation between the simulation and experimental results rises so that a relative error of 25.8% is observed between them. Therefore, normalizing each data set and calculating *P* value is an excellent way to compare the degree of conformity between results. Figure 6d indicates the normalized experimental and simulation data with a calculated *P* value of 0.328, indicating good conformity between them. The deviation between results can be attributed to microchannel surface roughness caused by laser engraving on polymer, leading to increased friction between surface and fluid. Subsequently, the flow regime inside the microchannel becomes a little bit turbulent. In contrast, in the simulation assumptions, the inner surface of the microchannel is considered completely smooth with hydrodynamic laminar flow.

**Figure 7:**
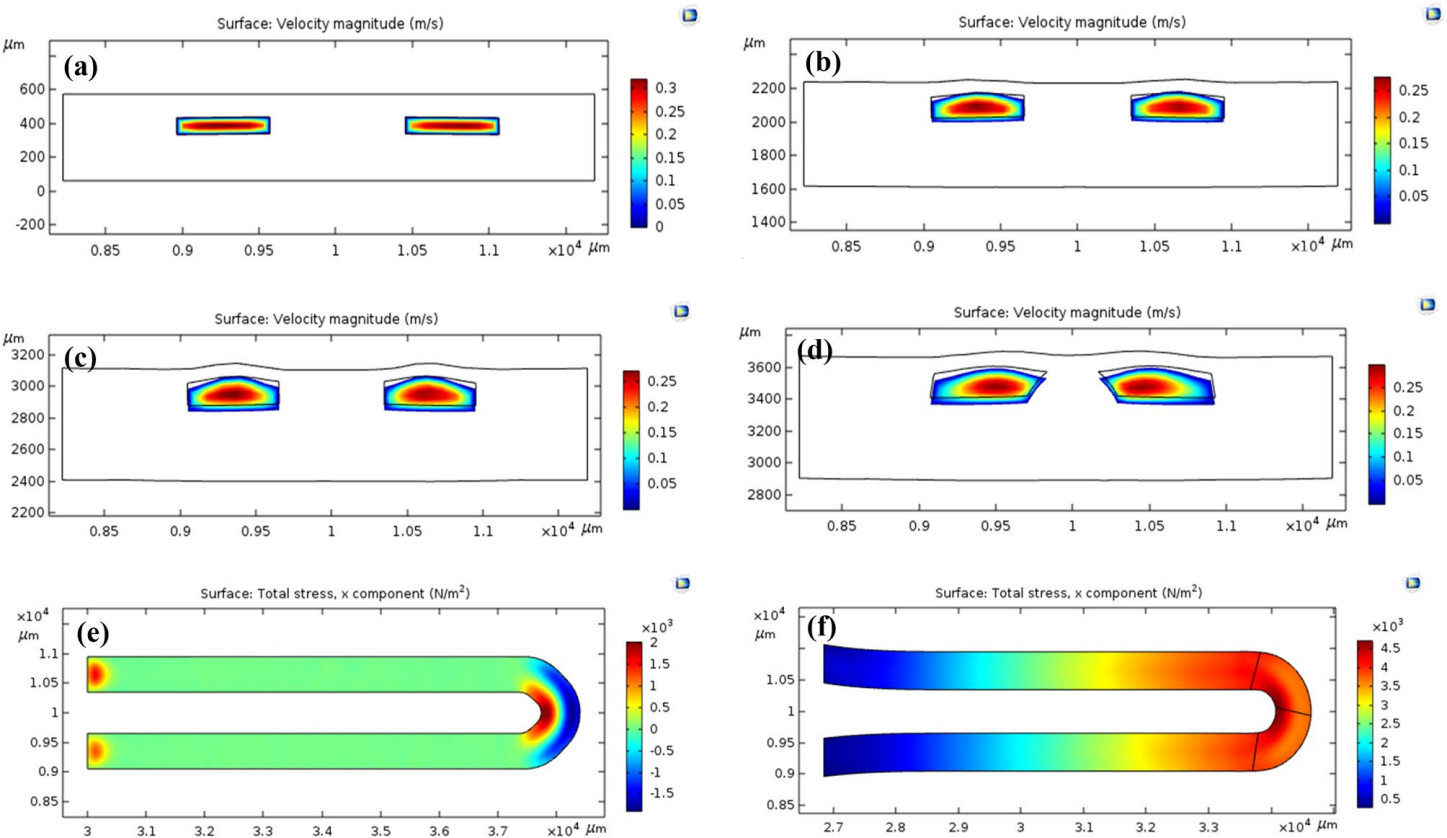
Two-dimensional velocity distribution with an input flow rate of 700 µl/min in a curved layer, longitudinal distance from the origin of coordinates (**a**) 30,000 µm, (**b**) 35,000 µm, (**c**) 36,000 µm and (**d**) 36,500 µm. Distribution of stress applied by fluid to the microchannel wall at the input flow rate of 600 µl/min. (**e**) Straight cantilever, and (**f**) curved cantilever. (COMSOL Multiphysics 5.3, https://www.comsol.com).

The fluid velocity distribution at certain cross-sections is shown in Fig. 7. According to the results obtained by the simulation at a flow rate of 700 µl/min, the flow hydrodynamic inside the microchannel is laminar, and no turbulency is observed along the flow path. At a given area of the cantilever, the maximum velocity was observed in the center of the microchannel, whose approximate value equals 0.3 m/s (Fig. 7a). Nevertheless, as the vertical cross-section changes, fluid velocity reduces due to the flexibility of the material. The momentum force resulting from this velocity change leads to cantilever deformation, as can be seen in Fig. 7b–d. Given that a cantilever is a type of pressure flowmeter whose performance relies on applied force, overall stress distribution must be studied. For this purpose, the distribution of fluid stress on the upper surface of the microchannel wall in a two-dimensional diagram for both straight and curved cantilevers is shown in Fig. 7e, f. According to the diagram, it is evident that the total stress applied by fluid will increase by making a curved cantilever. According to the results, the maximum shear stress belongs to change in the fluid direction, whose value is equal to 2000 and 4500 N/m^2^ in straight microcantilever and curved microcantilever, respectively. A fluid exerts stress on the microchannel walls in both normal and tangent directions. This stress emerges due to changes in velocity (shear stress) and pressure (normal stress) inside the microchannel. In a straight cantilever, stress exerted by fluid is tangent to the wall, while by making a curved cantilever, the stress is applied to the surface of the wall at a certain angle and causes the cantilever to push along the flow direction and bend.

## Conclusion

In this study, a suspended polymeric microfluidic system was fabricated to monitor the liquid flow rate in a microchannel. Prior to the fabrication process, a simulation analysis was performed to optimize the dimensions and other contributing factors. According to the simulation results, the larger the surface perpendicular to the flow, the higher rate of fluid will enter the cantilever, which causes the bending to increase. The fabricated polymeric suspended flowmeter with a sensitivity of 0.126 µm/(µl/min) measures flow rate within the range of 100–1000 µl/min. Ultimately, according to the comparison between the experimental results and simulation data, an acceptable consistency was obtained. The main result of this work refers to the curvature of the cantilever, stating that for accurate measuring, a curved structure is preferred to a straight one. Moreover, many conventional commercial flowmeters are not suitable for measuring low flow rates. Despite the low number of studies conducted in this field, the present paper was an attempt to provide a suitable flowmeter at the microfluidic scale. The advantages of the proposed flowmeter include its reasonable price and simple manufacturing method because of its simple structure consisting of only two thin layers of polymer and the laser engraving method required for its fabrication. High sensitivity and linear cantilever deflection based on different fluid flows are other advantages. The results were acceptable in terms of accuracy and repeatability, and an error of less than 2% was obtained. On the other hand, for its commercialization, a more appropriate detection method, such as resistance or capacitive technique, should be employed.
